# In Situ Synthesis of CoMoO_4_ Microsphere@rGO as a Matrix for High-Performance Li-S Batteries at Room and Low Temperatures

**DOI:** 10.3390/molecules29215146

**Published:** 2024-10-31

**Authors:** Ronggang Zhang, Haiji Xiong, Jia Liang, Jinwei Yan, Dingrong Deng, Yi Li, Qihui Wu

**Affiliations:** 1 Electronic and Mechanical Engineering, Fujian Polytechnic Normal University, Fuzhou 350300, China; 2 College of Marine Equipment and Mechanical Engineering, Key Laboratory of Energy Cleaning Utilization, Development, Cleaning Combustion and Energy Utilization Research Center of Fujian Province, Xiamen Key Laboratory of Marine Corrosion and Smart Protective Materials, Jimei University, Xiamen 361021, Chinadrdeng@jmu.edu.cn (D.D.); 3 Jiangsu Key Lab of Advanced Functional Polymer Design and Application, Department of Polymer Science and Engineering, College of Chemistry, Chemical Engineering and Materials Science, Soochow University, Suzhou 215123, China

**Keywords:** CoMoO_4_, lithium–sulfur batteries, cathode, low temperature

## Abstract

Lithium–sulfur batteries (Li-S batteries) have attracted wide attention due to their high theoretical energy density and the low cost of sulfur cathode material. However, the poor conductivity of the sulfur cathode, the polysulfide shuttle effect, and the slow redox kinetics severely affect their cycling performance and Coulombic efficiencies, especially under low-temperature conditions, where these effects are more exacerbated. To address these issues, this study designs and synthesizes a microspherical cobalt molybdate@reduced graphene oxide (CoMoO_4_@rGO) composite material as the cathode material for Li-S batteries. By growing CoMoO_4_ nanoparticles on the rGO surface, the composite material not only provides a good conductive network but also significantly enhances the adsorption capacity to polysulfides, effectively suppressing the shuttle effect. After 100 cycles at room temperature with a current density of 1 C, the reversible specific capacity of the battery stabilizes at 805 mAh g^−1^. Notably, at −20 °C, the S/CoMoO_4_@rGO composite achieves a reversible specific capacity of 840 mAh g^−1^. This study demonstrates that the CoMoO_4_@rGO composite has significant advantages in suppressing polysulfide diffusion and expanding the working temperature range of Li-S batteries, showing great potential for applications in next-generation high-performance Li-S batteries.

## 1. Introduction

With the rapid development of new energy technologies, high-energy-density and low-cost energy storage technologies have become crucial for advanced fields such as electric vehicles [1] and renewable energy storage [2,3]. Lithium–sulfur batteries (Li-S batteries), due to their extremely high theoretical energy density (2600 Wh kg^−1^) and the abundance of sulfur resources, demonstrate enormous potential in future high-efficiency energy storage devices [4,5]. The sulfur cathode material in Li-S batteries has a high theoretical specific capacity (1675 mAh g^−1^) [6], providing significant advantages over traditional lithium-ion battery cathode materials [7,8]. In addition, the low cost [9] and environmental friendliness of sulfur further enhance the attractiveness of Li-S batteries for large-scale applications [10,11].

However, the development of Li-S batteries still faces numerous challenges: first, sulfur itself has poor conductivity, limiting the rate capability and overall energy output [12]; secondly, soluble polysulfides (Li_2_S_n_, 2 ≤ *n* ≤ 8) formed during charging and discharging can diffuse into the electrolyte [13], causing the “shuttle effect”, which leads to rapid capacity decay and therefore reduces the Coulombic efficiency [14,15]. In addition, the volume expansion of the sulfur cathode also affects the structural stability of batteries [16,17]. Under extreme conditions, especially at low temperatures, these issues become even more severe. At low temperatures, the ionic diffusion rate in Li-S batteries decreases, the electrochemical reactions of electrode materials slow down, and the polarization becomes more pronounced, severely limiting their properties in cold climates [18,19]. Therefore, it is urgent to develop new cathode materials and corresponding battery systems to address the performance degradation in low-temperature environments [20]. To overcome the performance limitations of Li-S batteries under both room-temperature and low-temperature conditions, researchers have conducted extensive studies on the cathode, anode, and separator [21]. On the cathode side, using highly conductive materials to support sulfur or introducing materials with polysulfide adsorption capability can effectively suppress the shuttle effect and improve the conductivity [22,23]. On the anode side, researchers have improved anode stability and reduced lithium dendrite formation through surface modifications and alloy anode materials [24,25]. Additionally, by incorporating polysulfide adsorbents or functionalized separators in the electrolyte or separator, the shuttle effect can be further reduced, enhancing the batteries’ cycling performance [26,27].

Although these methods have improved the performance of Li-S batteries to some extent, they each have certain limitations. In the anode protection strategy, the dendrite problem can be improved by surface modification and lithium alloyed anodes (such as lithium silicon, lithium tin alloys), but the electrochemical reaction performance of these materials is usually poor at low temperatures, and the electrode structure may be destroyed due to volume expansion, which will affect the life and efficiency of the battery. Although the functionalized diaphragm can effectively reduce the shuttle effect of LiPS, its adsorption capacity may be decreased under long or high-rate cycling, especially in low-temperature environments, the structural stability and ion conductivity of the diaphragm will be greatly affected. In contrast, addressing Li-S battery issues from the perspective of cathode materials offers unique advantages. Improvements on the cathode side can not only effectively enhance the conductivity of sulfur but also suppress the shuttle effect by designing appropriate polysulfide adsorption materials while also increasing the structural stability of the electrode. Moreover, optimizing cathode materials has a direct and long-term effect on the overall performance of the batteries, especially under low-temperature conditions, where superior cathode materials can maintain good electrochemical activity across a wide temperature range. In recent years, many researchers have developed various sulfur-based cathode composites aimed at improving the electrochemical performance of Li-S batteries. For example, carbon-based materials (such as carbon nanotubes, graphene, porous carbon, etc.) in combination with sulfur can significantly improve conductivity and sulfur-loading capacity [28,29]. However, although carbon materials exhibit good conductivity, they cannot effectively adsorb polysulfides, making it difficult to completely suppress the shuttle effect. To address the diffusion of polysulfides, researchers have introduced compounds such as metal oxides and sulfides, including TiO_2_and MnO_2_ [30,31], utilizing their abundant active sites to adsorb polysulfides. However, these materials generally suffer from low conductivity, requiring further optimization to achieve better performance in practical applications.

In response to the above issues, this study proposes a novel cobalt molybdate (CoMoO_4_)@reduced graphene oxide (rGO) composite material as the main cathode material for Li-S batteries. CoMoO_4_, as a transition metal oxide, has high electrocatalytic activity and strong polysulfide adsorption capacity, which can effectively suppress the shuttle effect and enhance the electrochemical stability of the sulfur cathode [32]. In addition, CoMoO₄ has relatively high electrical conductivity, enhancing the conductivity of the electrode material and addressing the poor conductivity of traditional metal oxide materials. On the other hand, rGO has excellent conductivity and a high specific surface area, providing an ideal platform for CoMoO_4_ loading and forming a three-dimensional conductive network, which helps further improve sulfur conductivity. By growing CoMoO_4_ on rGO, the composite material in this study can not only suppress the diffusion of polysulfides but also provide excellent electrochemical performance at both room and low temperatures.

## 2. Results

**Figure 1:**
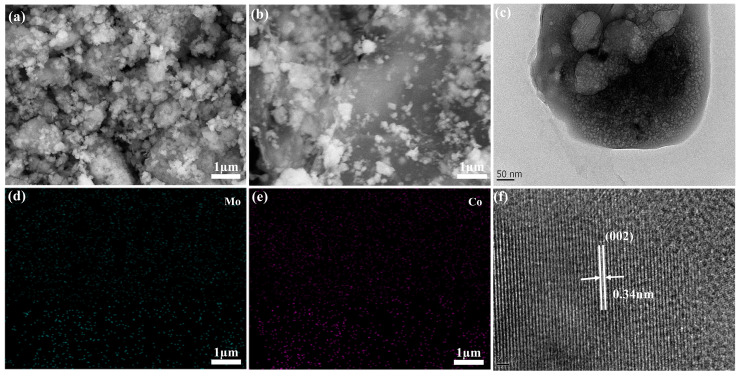
(**a**) SEM image of the CoMoO₄ sample, (**b**) SEM image of the CoMoO₄@rGO sample, (**c**) TEM image, (**d**,**e**) elemental mapping images, (**f**) HRTEM image.

CoMoO_4_@rGO was synthesized in situ using a self-templating method, and the prepared CoMoO_4_@rGO composite was characterized and analyzed using scanning electron microscopy (SEM) and transmission electron microscopy (TEM). As shown in the SEM image in Figure 1a, when CoMoO_4_ particles are exposed to a reactive environment with a high surface energy, the particles will spontaneously tend to reduce the total surface energy, reducing the surface area by agglomerating with each other. As a result, CoMoO₄ particles exhibit severe agglomeration with sizes ranging from 0.3 to 3 μm, appearing as microspheres. The introduction of rGO matrix allows CoMoO₄ to grow uniformly on the rGO surface (Figure 1d,e), with no obvious agglomeration, and the particle size distribution is between 100 and 300 nm (Figure 1b). In higher-magnification SEM images (Figure S1), the microspherical structure of CoMoO_4_@rGO is seen to be composed of numerous primary nanoparticles aggregated into larger particles. This structure provides the microspheres with numerous pores, significantly enlarging the catalytic surface for lithium polysulfide (LiPS) redox reactions. In the TEM image, these primary nanoparticles can be seen more clearly (Figure 1c). The high-resolution TEM (HRTEM) image (Figure 1f) shows clear lattice fringes in the prepared CoMoO_4_@rGO, with an interplanar spacing of 0.34 nm, which corresponds well to the (002) lattice plane of CoMoO_4_ (PDF #210868).

**Figure 2:**
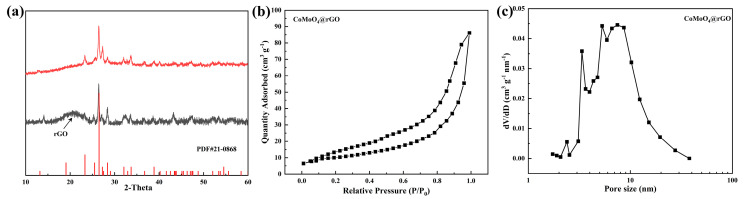
(**a**) XRD pattern of CoMoO₄@rGO (black) and CoMoO₄ (red), and (**b**,**c**) nitrogen adsorption–desorption isotherms of the CoMoO₄@rGO and pore size distribution.

The crystal structure of the molybdate was further confirmed by X-ray diffraction (Figure 2a). Clearly, the obtained XRD pattern matches well with the standard pattern of CoMoO_4_ (PDF #210868), consistent with the reports in the literature [33]. The structural properties of CoMoO_4_@rGO can also be assessed through N_2_ adsorption–desorption isotherms. As shown in Figure 2b, the prepared CoMoO_4_@rGO exhibits a typical type IV isotherm and H_3_-type hysteresis loop, indicating the presence of mesoporous and slit-like pores. The capillary condensation observed during high-pressure adsorption is related to mesopore adsorption behavior, while the loop reflects the slit-like pores formed by the aggregation of CoMoO_4_ particles. For CoMoO_4_@rGO and CoMoO_4_, their specific surface areas are 165.9 and 30.3 m^2^ g^−1^, respectively (Figure S2a). Such a significant difference is due to the better dispersion and smaller particle size of CoMoO₄ in CoMoO₄@rGO, which provides abundant anchoring sites for LiPSs. Figure 2c shows that the pore size distributions of CoMoO_4_@rGO and CoMoO_4_ are mainly concentrated in the mesoporous structure of 2 to 10 nm (Figure S2b). However, CoMoO_4_@rGO has a larger pore volume of 0.48 cm^3^ g^−1^, while that of CoMoO₄ is only 0.06 cm^3^ g^−1^. The larger pore volume is beneficial for alleviating the volume expansion caused during the charging and discharging processes of the battery, which is crucial for extending the battery’s lifespan.

**Figure 3:**
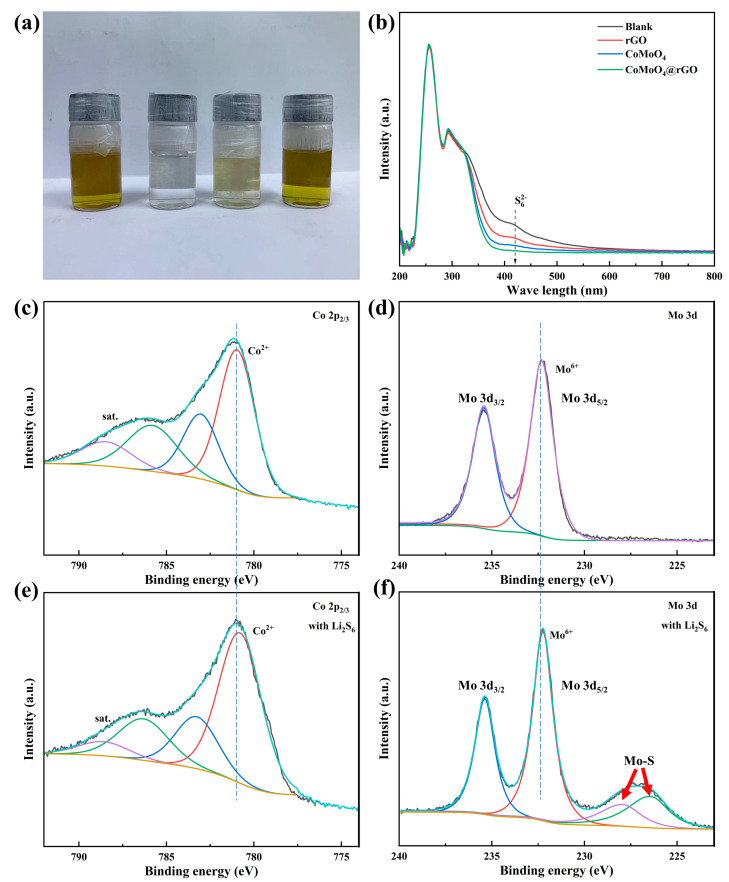
(**a**) The visual distinction and (**b**) UV−visible spectrum of Li_2_S_6_ solution after adding equal amounts of CoMoO_4_@rGO, CoMoO_4_, and rGO and letting it sit for 12 h. Core spectra of (**c**,**e**) Co 2p2/3 and (**d**,**f**) Mo 3d before and after treatment with Li_2_S_6_.

**Figure 4:**
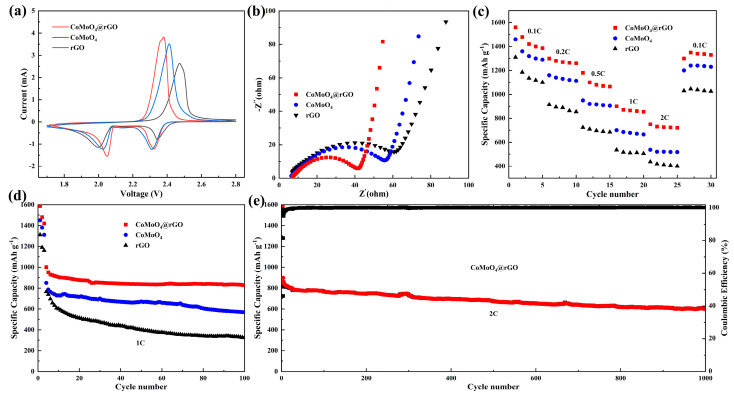
(**a**) Cyclic voltammetry tests of S/CoMoO_4_@rGO, S/CoMoO_4_, and S/rGO cathodes at a scan rate of 0.1 mV s^−1^ at room temperature; (**b**) Nyquist plot before cycling; (**c**) rate performance at different current densities; (**d**) the relationship between discharge capacity and cycle number at a current density of 1 C; (**e**) the relationship between charge−discharge capacity, coulombic efficiency, and cycle number at a current density of 2 C.

To compare the materials’ adsorption capacity for LiPSs, adsorption experiments and UV–visible measurements were conducted. Specifically, equal amounts of CoMoO₄@rGO, CoMoO_4_, and rGO were added to the same volume of Li_2_S_6_ solution (3 mmol L^−1^). The photos of the solutions after standing for 12 h are shown in Figure 3a. It can be observed that the solution with rGO shows little color change, whereas the solutions with CoMoO_4_@rGO and CoMoO_4_ exhibit more significant discoloration, with the CoMoO_4_@rGO solution becoming nearly transparent. This indicates that CoMoO_4_@rGO has a stronger adsorption capacity for LiPSs compared to CoMoO_4_. This is also confirmed by UV–visible (UV-vis) absorption spectra (Figure 3b), where the characteristic peak of Li_2_S_6_ in the solution soaked with CoMoO_4_@rGO completely disappeared. Furthermore, during the experiment, CoMoO_4_@rGO almost completely lost its color within 30 min, demonstrating a strong interaction between CoMoO_4_@rGO and LiPSs. From the weak characteristic peak of Li_2_S_6_, it can be observed that CoMoO_4_’s adsorption capacity for LiPS is slightly inferior, while the Li_2_S_6_ solution with rGO shows little color change and retains stronger characteristic peaks of Li_2_S_6_. The sample Mo 3d and Co 2p_2_/_3_ core spectra before and after LiPS adsorption were studied using XPS, revealing the chemical interactions between LiPSs and CoMoO_4_@rGO. In the original CoMoO_4_@rGO, Co is in a divalent state. After interaction with Li_2_S_6_, the binding energy of Co^2+^ shows a certain negative shift, indicating the formation of metal–sulfur bonds. For the Mo 3d spectrum of CoMoO₄@rGO, Mo is in the Mo (VI) oxidation state, with a pair of Mo 3d_5_/_2_ and Mo 3d_3_/_2_ peaks present. After interaction with Li_2_S_6_, in addition to a small shift of the Mo⁶⁺ peak towards lower binding energy, a pair of new 3d_5_/_2_ and 3d_3_/_2_ peaks appears at lower binding energies (226.80 and 228.74 eV), corresponding to the newly formed Mo-S bonds (Figure 4c). XPS spectra and adsorption experiments indicate that CoMoO_4_@rGO mainly forms metal–sulfur bonds through Co and Mo with sulfur, as well as forming Mo-S bonds that interact strongly with Li_2_S_6_, which inhibited the shuttle effect inside the Li-S batteries. This strong adsorption will be beneficial to the catalytic conversion of LiPSs.

To investigate the effect of the S/CoMoO_4_@rGO cathode on the electrochemical performance of Li-S batteries, S/CoMoO_4_@rGO, S/CoMoO_4_, and S/rGO composite materials were synthesized using the melt diffusion method and assembled into Li-S batteries for testing. Firstly, cyclic voltammetry (CV) tests were conducted on these three batteries, as shown in Figure 4a. It can be observed that all three Li-S batteries assembled with different cathodes exhibit one oxidation peak and two reduction peaks. However, the peak potentials differ among the three cathodes: the oxidation peaks correspond to potentials of 2.49 V, 2.42 V, and 2.39 V, while the first reduction peaks correspond to 2.35 V, 2.31 V, and 2.32 V, and the second reduction peaks correspond to 2.00 V, 2.02 V, and 2.06 V, respectively. The addition of the rGO matrix enhanced the conductivity of the sulfur cathode and allowed for a more uniform distribution of CoMoO_4_ particles. As a result, the S/CoMoO_4_@rGO cathode exhibits the highest peak current and the smallest potential difference, indicating that S/CoMoO_4_@rGO facilitates the redox kinetics of Li-S batteries. Subsequently, electrochemical impedance spectroscopy (EIS) measurements were conducted on the batteries assembled with the three cathodes, as shown in Figure 4b. The batteries exhibited an ohmic resistance (R_0_) of approximately 7 Ω; however, the charge transfer resistances (Rct) for S/CoMoO_4_@rGO, S/CoMoO_4_, and S/rGO were 53 Ω, 48 Ω, and 35 Ω, respectively. This result indicates that smaller CoMoO_4_ particular sizes and more uniform dispersion are beneficial for increasing the contact area with the electrolyte, thereby reducing the transfer resistance of electrons/ions within the battery. At low temperatures, the viscosity of the electrolyte significantly increases due to the low temperature, which slows down the kinetic processes in the battery. Consequently, the capacity of Li-S batteries drastically decreases or may even fail to operate normally at low temperatures. From the low-frequency region, it can also be observed that the S/CoMoO_4_@rGO cathode has the lowest ionic diffusion resistance compared to the S/CoMoO_4_ and S/rGO cathodes, suggesting that Li-S batteries based on the S/CoMoO_4_@rGO cathode that should also perform well at low temperatures. Figure S3 shows the initial charge–discharge curves of Li-S batteries assembled with S/CoMoO_4_@rGO, S/CoMoO_4_, and S/rGO cathodes under a current density of 0.1 C. It can be observed that the battery containing S/CoMoO_4_@rGO exhibits a specific capacity of 1562 mAh g^−1^, whereas the S/CoMoO_4_ and S/rGO cathodes only release capacities of 1458 and 1310 mAh g^−1^, respectively, indicating that CoMoO_4_@rGO indeed enhances the reaction kinetics of LiPSs conversion. Moreover, when the nominal specific capacity is approximately 800 mAh g^−1^, the potential differences ΔE between the discharge and charge curves for the three cathodes are 150 mV, 165 mV, and 174 mV, respectively, further indicating that Li-S batteries based on the S/CoMoO_4_@rGO cathode exhibit the lowest polarization and the best reversibility. Figure 4c illustrates the initial charge–discharge curves of Li-S batteries with S/CoMoO_4_@rGO, S/CoMoO_4_, and S/rGO cathodes at different current densities. At current rates of 0.1, 0.2, 0.5, 1, and 2.0 C, the reversible discharge capacities of the Li-S batteries assembled with the three cathodes are 1562, 1458, and 1310 mAh g^−1^; 1308, 1157, and 915 mAh g^−1^; 1179, 953, and 720 mAh g^−1^; 907, 699, and 534 mAh g^−1^; and 754, 530, and 438 mAh g^−1^, respectively. At these various current densities, the S/CoMoO_4_@rGO cathode demonstrates a significant advantage, indicating its excellent rate performance. To ensure the long-term effective use of the battery, cycling stability is also an important criterion. As shown in Figure 4d, after 100 cycles at a current density of 1 C, the batteries with S/CoMoO_4_@rGO, S/CoMoO_4_, and S/rGO cathodes maintain capacities of 827 mAh g^−1^, 568 mAh g^−1^, and 320 mAh g^−1^, respectively, with capacity retention rates of 82.7%, 67%, and 42%. Furthermore, the Li-S battery with the S/CoMoO_4_@rGO cathode maintains a reversible specific capacity of 602 mAh g^−1^ after 1000 cycles at a current density of 2 C, with an average capacity decay of only 0.026% per cycle (Figure 4e). The S/CoMoO_4_@rGO cathode also has a capacity retention rate of 44% at a current density of 5 C (Figure S4). The results indicate that the batteries assembled with S/CoMoO_4_@rGO exhibit good long-cycle stability at room temperature. Because the strong interaction between CoMoO_4_ polar bonds and LiPSs can effectively adsorb and catalyze the conversion of LiPSs, the cell containing the CoMoO_4_-coated functional cathode can effectively inhibit the shuttle effect of LiPSs. Compared with CoMoO_4_@rGO, CoMoO_4_ does not significantly improve the battery performance of lithium–sulfur batteries due to serious agglomeration, general conductivity, and limited adsorption sites. In addition, the introduction of the rGO matrix strengthens the conductive network of the electrode, thereby reducing charge transfer resistance and significantly enhancing the discharge capacity, rate capability, and cycling stability of the Li-S battery.

**Figure 5:**
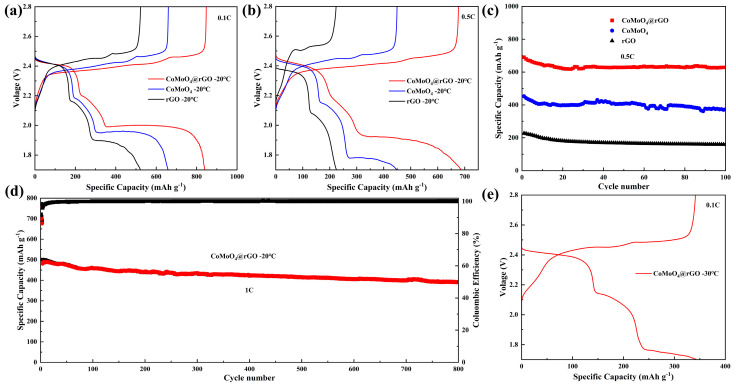
(**a**) Charge−discharge curves of S/CoMoO_4_@rGO, S/CoMoO_4_, and S/rGO cathodes at 0.1 C at −20 °C. (**b**) Charge−discharge curves at 0.5 C. (**c**) The relationship between discharge capacity and cycle number at a current density of 0.5 C. (**d**) The relationship between charge−discharge capacity, coulombic efficiency, and cycle number at a current density of 1 C. (**e**) Charge−discharge curves of S/CoMoO_4_@rGO cathodes at 0.1 C at −30 °C.

The applications of Li-S batteries in low-temperature environments face numerous challenges, such as the increasing activation energy under low-temperature conditions, which severely hinders the conversion process of LiPSs [34,35]. Additionally, the viscosity of the electrolyte increases [36], resulting in a decrease in the dissociation degree of lithium salts and consequently reducing the ionic conductivity of the electrolyte [36,37,38]. These issues will lead to a significant decline in performance, primarily manifested as capacity degradation, reduced rate capability, and shortened cycle life of the batteries. To address the difficulty of Li-S batteries in low-temperature scenarios, further electrochemical performance studies were conducted at −20 °C. Figure 5a shows the charge–discharge curves at −20 °C and a current density of 0.1 C, where the reversible specific capacities of the Li-S batteries based on S/CoMoO_4_@rGO, S/CoMoO_4_, and S/rGO cathodes are 840, 657, and 516 mAh g^−1^, respectively. Moreover, the third discharge platform potentials for the three cathodes at low temperatures are approximately 1.98 V, 1.95 V, and 1.89 V, indicating that the S/CoMoO_4_@rGO cathode exhibits the least battery polarization, which corresponds to the charge–discharge tests conducted at room temperature. When the current density is further increased to 0.5 C, the reactants are rapidly consumed, and the reactants in the electrolyte (such as lithium ions or polysulfides) need to diffuse to the surface of the electrode. However, diffusion is a slow process at low temperatures. With the increase in current density, in order to maintain a high reaction rate, the battery needs to apply a higher voltage, which causes the polarization effect of the battery to be more serious [39]. For these reasons, it is nearly impossible for lithium–sulfur batteries based on S/rGO cathodes to perform liquid–solid reactions at a current density of 0.5 C, resulting in a capacity release of less than 230 mAh g^−1^. Due to the severe aggregation of CoMoO_4_, its own conductive network is poor due to the lack of rGO matrix introduction. While the S/CoMoO_4_ cathode exhibits good performance at room temperature, its performance is less than ideal at low temperatures, with the third platform dropping below 1.79 V. After 100 cycles, the battery only has a reversible specific capacity of 377 mAh g^−1^. Conversely, the S/CoMoO_4_@rGO cathode, which possesses a robust conductive network and strong adsorption capacity for LiPSs, plays a very effective catalytic role in the reaction process of Li-S batteries, significantly enhancing the kinetic performance and reducing battery polarization. Its third voltage platform remains around 1.91 V, and it releases a reversible specific capacity close to 680 mAh g^−1^, which is almost more than three times that of the S/rGO cathode. After 100 cycles, the battery can still deliver a reversible specific capacity of 628 mAh g^−1^ at −20 °C, with an average capacity decay of only 0.08% per cycle. Even at a larger current density of 1 C, the S/CoMoO_4_@rGO cathode can still maintain a capacity release of over 485 mAh g^−1^. Furthermore, after 800 cycles, the capacity can be maintained above 390 mAh g^−1^, which is a very commendable level for low-temperature lithium–sulfur batteries. At −30 °C, the S/CoMoO_4_@rGO cathode can also facilitate the liquid–solid reaction at the third platform, with a capacity release exceeding 340 mAh g^−1^ in the first cycle. This further demonstrates that the cathode prepared with CoMoO_4_ nanoparticles grown in situ on the rGO matrix exhibits good conductivity and excellent ionic diffusion capability, improving the reaction kinetics under low-temperature conditions in lithium–sulfur batteries. Compared with other advanced cathode materials used at low temperatures (Table S1), the CoMoO_4_@rGO cathode also has obvious advantages in long-cycle stability and capacity release [40,41,42,43,44,45,46,47,48,49].

## 3. Materials and Methods

### 3.1. Synthesis of S/CoMoO_4_@rGO Composites

Dissolve 4 mmol of cobalt nitrate hexahydrate in 40 mL of isopropanol, stir evenly, then slowly add 12 mL of glycerol dropwise to the above solution. After stirring for 30 min, transfer the solution to an autoclave, heat it to 180 °C in a drying oven, and keep it at that temperature for 6 h. This step is to synthesize cobalt-based nanoprecursors, and the solvothermal reaction at 180 °C and 6 h is to control the morphology and particle size of the final cobalt-based microspheres. After the reaction, centrifuge the product, wash it with deionized water three times, and dry it at 60 °C for 12 h in a vacuum drying oven. Dissolve 2 mmol of sodium molybdate, 40 mg of reduced graphene oxide, and 80 mg of the above product in 40 mL of deionized water. After ultrasonic dispersion, transfer the solution to an autoclave and heat it to 120 °C in an oven, maintaining the temperature for 6 h. The temperature of 120 °C is sufficient to drive the reaction of sodium molybdate with the cobalt precursor to form CoMoO_4_, but it is not sufficient to cause large particle agglomeration or phase transition, ensuring that the morphology of the material is controlled. The 6 h time provides sufficient time for the reaction to ensure uniform binding of the precursor particles and rGO matrix to form a stable precursor substance. This also provides a more uniform and detailed structural basis for subsequent high-temperature calcination steps. After the reaction, centrifuge the product, wash it with deionized water three times, and dry it at 60 °C for 12 h in a vacuum drying oven. Then, the dried material was calcined in a tube furnace for 3 h in a nitrogen atmosphere at a heating rate of 5 °C min^−1^ to obtain microspherical CoMoO_4_@rGO. High-temperature calcination at 500 °C can promote the close combination of nano-scale CoMoO_4_ and rGO to form a stable composite structure, and can effectively remove residual organic matter or impurities. The preparation method of CoMoO_4_ is the same as that of CoMoO_4_@rGO, except that no rGO is added. The S/CoMoO_4_@rGO composite material is prepared by a simple melt diffusion method, where the sulfur and CoMoO_4_ mixture is heated at 155 °C for 12 h in a drying oven, fully ground, sieved, and then used. Commercial rGO is used as a comparison substrate. S/CoMoO_4_ and S/rGO composites are prepared using the same melt diffusion method.

### 3.2. Polysulfide Adsorption Sample Preparation

Mix Li_2_S and S (molar ratio 1:5) in 1,3-dioxolane/ethylene glycol dimethyl ether (DOL/DME, volume ratio 1:1) and heat at 60 °C while stirring for 48 h to obtain a Li_2_S_6_ solution. Then, equal masses of CoMoO_4_@rGO, CoMoO_4_, and rGO are added to the prepared Li_2_S_6_ solution separately. Observe the color change of the solution and test the ultraviolet–visible absorption spectrum of the supernatant. The remaining solids are dried overnight for X-ray photoelectron spectroscopy (XPS) testing.

### 3.3. Electrochemical Measurements

Dissolve 70 wt% active composite material, 20 wt% conductive agent (Super P), and 10 wt% binder (LA 5%) in a water–alcohol mixture. After ball-milling for 12 h, the slurry is coated onto aluminum foil to prepare the cathode. The area of the aluminum foil is 13 mm and the sulfur loading is 1.2–1.5 mg cm^−2^. The electrolyte consists of 0.5 M LiCF_3_SO_3_ and 0.5 M LiNO₃ (dissolved in 1,2-dimethoxyethane and dioxolane in a 1:1 volume ratio, 35 μL). Battery cycling tests were conducted using a NEWARE BTS-5 V/20 mA battery tester (Shenzhen, China) with a voltage window of 1.7–2.8 V at room temperature. Cycling performance was tested at different rates (1 C = 1675 mA g^−1^). The specific capacity was calculated based on sulfur as the active material. Cyclic voltammetry measurements and EIS data collection were carried out on a DH7000C workstation, with a frequency range of 0.1 Hz to 10 kHz.

## 4. Conclusions

In summary, a novel microspherical CoMoO_4_@rGO composite material was prepared and used as the main cathode material for Li-S batteries. The incorporation of the rGO matrix enhanced the conductive network of the electrode, and the in situ growth of CoMoO_4_ nanoparticles on rGO ensured a sufficiently uniform distribution of reactive sites. Additionally, since the microspherical CoMoO_4_ is composed of numerous aggregated CoMoO_4_ ions, each microsphere is sufficiently porous. A comparison of Li-S batteries assembled with CoMoO_4_@rGO and control materials yielded the following main findings:

1. The CoMoO_4_@rGO composite material was synthesized using a solvothermal method followed by low-temperature annealing. The microspherical structure significantly alleviates volume expansion and damage to the electrode during the charge–discharge process. Compared to CoMoO_4_, the introduction of rGO reduces the size of CoMoO_4_ microspheres, indirectly increasing the specific surface area of CoMoO_4_. The increased number of adsorption sites compared to the control materials significantly suppresses the shuttling effect.
2. At room temperature, the S/CoMoO_4_@rGO cathode exhibits the least battery polarization compared to the control materials. The electrochemical impedance tests also reveal the smallest charge transfer resistance and the best ionic diffusion rate. This outstanding performance accelerates the conversion of LiPS, resulting in a reversible specific capacity of 1562 mAh g^−1^ for the battery at 0.1 C. After cycling at a density of 2 C for 1000 cycles, it also exhibits a capacity decay rate of 0.0026%.
3. At low temperatures, the lithium–sulfur battery based on the S/CoMoO_4_@rGO cathode exhibits good cycling stability. Furthermore, when the temperature drops to −30 °C, the battery can still perform the liquid–solid reaction at the third platform. Data obtained at room and low temperatures indicate that the CoMoO_4_@rGO composite material is an excellent cathode material for lithium–sulfur batteries. Relative to the studies on battery systems at room temperature, the research on the low-temperature performance of lithium–sulfur batteries is currently not extensive. This provides a new perspective for the selection of cathode materials for lithium–sulfur batteries.

## Data Availability

Data are contained within the article and Supplementary Materials.

## Supplementary Materials

The following supporting information can be downloaded at https://www.mdpi.com/article/10.3390/molecules29215146/s1: Figure S1: SEM image of the CoMoO₄@rGO sample; Figure S2: (a) nitrogen adsorption–desorption isotherms of the CoMoO₄@rGO and (b) pore size distribution; Figure S3: Charge–discharge curves of S/CoMoO4@rGO, S/CoMoO_4_, and S/rGO cathodes at 0.1 C at room temperature; Figure S4. The relationship between charge-discharge capacity, coulombic efficiency, and cycle number at a current density of 5 C; Table S1. Comparison of low-temperature performance of lithium sulfur batteries with other studies. Refs. [40,41,42,43,44,45,46,47,48,49] are cited in Supplementary Materials.
