# Supplementary material for: In Situ Synthesis of CoMoO4 Microsphere@rGO as a Matrix for High-Performance Li-S Batteries at Room and Low Temperatures
Source: Molecules. 2024 Oct 31;29(21):5146. doi: 10.3390/molecules29215146 (PMC11547999; doi:10.3390/molecules29215146)
Supplement: Supplementary file 1 [file molecules-29-05146-s001.zip › molecules-3271800-supplementary.pdf]

Supporting Information

In Situ Synthesis of $CoMoO_4$ Microsphere@rGO as a Matrix for High-Performance Li-S Batteries at Room and Low Temperatures

Ronggang Zhang [1], Haiji Xiong [2], Jia Liang [2], Jinwei Yan [2], Dingrong Deng [2], Yi Li [3,*] and Qihui Wu [2,*]

[1] Electronic and Mechanical Engineering, Fujian Polytechnic Normal University, Fuzhou 350300, China
[2] College of Marine Equipment and Mechanical Engineering, Key Laboratory of Energy Cleaning Utilization, Development, Cleaning Combustion and Energy Utilization Research Center of Fujian Province, Xiamen Key Laboratory of Marine Corrosion and Smart Protective Materials, Jimei University, Xiamen 361021, China
[3] Jiangsu Key Lab of Advanced Functional Polymer Design and Application, Department of Polymer Science and Engineering, College of Chemistry, Chemical Engineering and Materials Science, Soochow University, Suzhou 215123, China
* Correspondence: liyi@suda.edu.cn (Y.L.); qihui.wu@jmu.edu.cn (Q.W.)

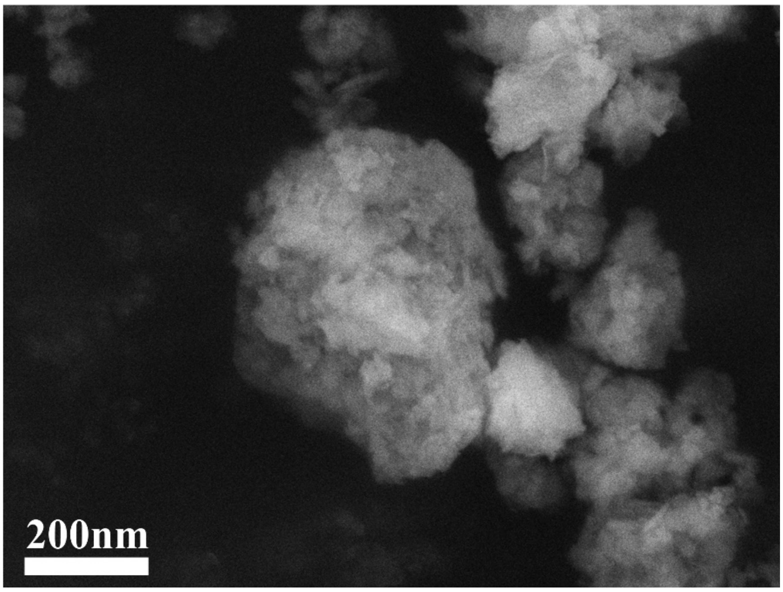

Figure S1. SEM image of the $CoMoO_4$@rGO sample.

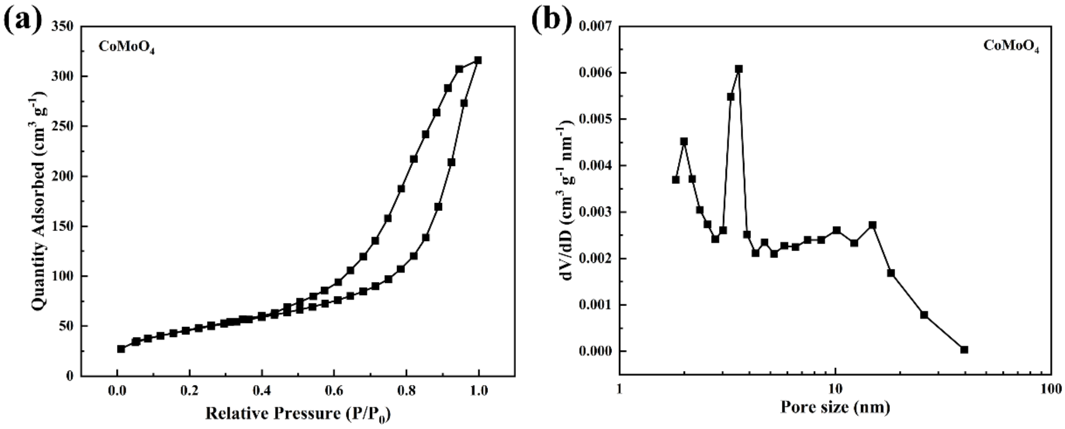

Figure S2. (a) nitrogen adsorption-desorption isotherms of the $CoMoO_4$@rGO and (b) pore size distribution.

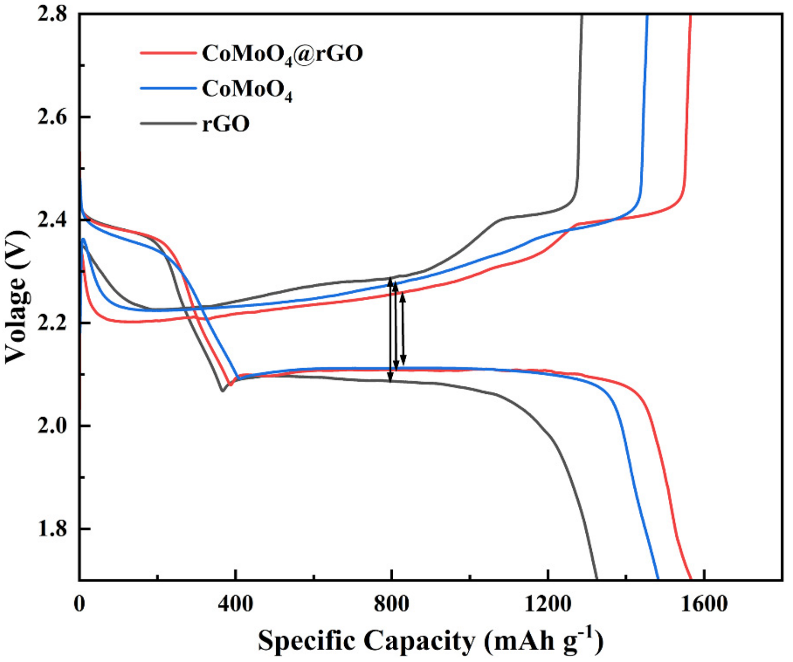

Figure S3. Charge-discharge curves of S/$CoMoO_4$@rGO, S/$CoMoO_4$, and S/rGO cathodes at 0.1 C at room temperature.

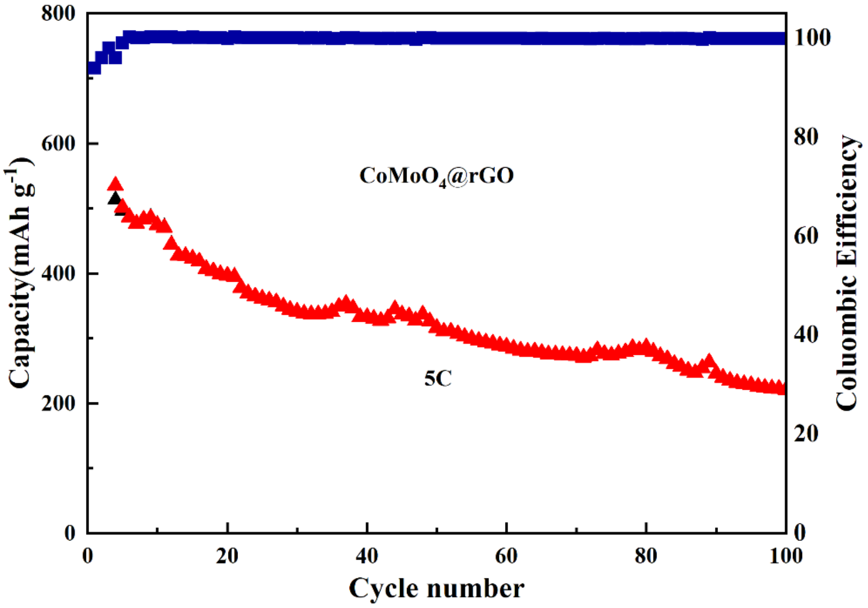

Figure S4. The relationship between charge-discharge capacity, coulombic efficiency, and cycle number at a current density of 5 C.

Table S1. Comparison of low temperature performance of lithium sulfur batteries with other literatures.

	Cathode	T (°C)	Capacity (mA h g^{-1})	Cycling	Current density	Ref.
1	carbon nanofibres@MnS	0	853	100	0.5 C	[s1]
2	$MoSe_2$ nanosheets	-25	252.8	500	1 C	[s2]
3	Co_3S_4@CNT	-25	740	200	0.1 C	[s3]
4	Fe_3C@foam carbon	-10	5.0 mAh cm^{-2}	100	0.1 C	[s4]
5	zirconium-based MOFs	-10	810	20	0.1 C	[s5]
6	CoFe@CNFs	0	700	5	0.1 C	[s6]
7	CoFe@C@CNFs	-10	≈900	80	0.5 C	[s7]
8	GO-Zn(II)-AmTZ	-20	310	100	0.5 C	[s8]
9	NiCo-MoOx/rGO	-20	518	100	0.5 C	[s9]
10	In_2O_3@NC-Co_3O_4	-20	755	100	0.1 C	[s10]
1	$CoMoO_4$@rGO	-20<br>-30	390<br>340	800<br>initial	1 C<br>0.1 C	This paper

References

[s1] Wang, X.; Zhao, X.; Ma, C.; Yang, Z.; Chen, G., Wang, L.; Sun, Z. Electrospun carbon nanofibers with MnS sulfiphilic sites as efficient polysulfide barriers for high-performance wide-temperature-range Li-S batteries. *J. Mater. Chem. A* **2020**, *8*, 1212-1220.

[s2] Fan, C.; Zheng, Y.; Zhang, X.; Shi, Y.; Liu, S.; Wang, H.; Zhang, J. High-performance and low-temperature lithium-sulfur batteries: synergism of thermodynamic and kinetic regulation. *Adv. Energy Mater.* **2018**, *8*, 1703638.

[s3] Wang, Y.; Xu, Y.; Ma, S.; Duan, R.; Zhao, Y.; Zhang, Y.; Li, C. Low temperature performance enhancement of high-safety lithium-sulfur battery enabled by synergetic adsorption and catalysis. *Electrochim. Acta* **2020**, *353*, 136470.

[s4] Zeng, P.; Yuan, C.; An, J.; Yang, X.; Cheng, C.; Yan, T.; Sun, X. Achieving reversible precipitation-decomposition of reactive Li_2S towards high-areal-capacity lithium-sulfur batteries with a wide-temperature range. *Energy Stor. Mater.* **2022**, *44*, 425-432.

[s5] Baumann, D.A.; Bennett A.E.; Díaz, K.J.; Thoi, V.S. Chemical Sulfide Tethering Improves Low-Temperature Li-S Battery Cycling. *ACS Appl. Mater. Interfaces* **2021**, *13*, 50862-50868.

[s6] Gao, N.; Zhang, Y.; Chen, C. Low-temperature Li-S battery enabled by CoFe bimetallic catalysts. *J. Mater. Chem. A* **2022**, *10*, 8378-8389.

[s7] Gao, N.; Li, B.; Li, X.; Zhao, J.; Wang, B. CoFe alloy-decorated interlayer with a synergistic catalytic effect improves the electrochemical kinetics of polysulfide conversion. *ACS Appl. Mater. Interfaces* **2021**, *13*, 57193-57203.

[s8] Zhang, Z.; Wang, Y.; Liu, J.; Sun, D.; Ma, X.; Jin, Y.; Cui, Y. A multifunctional graphene oxide-Zn (II)-triazole complex for improved performance of lithium-sulfur battery at low temperature. *Electrochim. Acta* **2018**, *271*, 58-66,

[s9] Chen, J.; Lei, J.; Zhou, J.; Chen, X.; Deng, R.; Qian, M.; Wu, F. Polysulfides adsorption and catalysis dual-sites on metal-doped molybdenum oxide nanoclusters for Li-S batteries with wide operating temperature. *Nano Res.* **2024**.

[s10] Wang, T., Wang, F., Shi, Z., Cui, S., Zhang, Z., Liu, W., & Jin, Y. Synergistic Effect of In_2O_3/NC-Co_3O_4 Interface on Enhancing the Redox Conversion of Polysulfides for High-Performance Li-S Cathode Materials at Low Temperatures. *ACS Appl. Mater. Interfaces* **2024**, *16*, 31158-31170.
